# Leptomeningeal Carcinomatosis From Primary Mucinous Carcinoma of the Ovary

**DOI:** 10.7759/cureus.51556

**Published:** 2024-01-02

**Authors:** Alexander Dye, Rachel Stein, Gregory Lewis, Karina Hew

**Affiliations:** 1 Obstetrics and Gynecology, University of Florida College of Medicine – Jacksonville, Jacksonville, USA; 2 Radiology, University of Florida College of Medicine – Jacksonville, Jacksonville, USA; 3 Gynecologic Oncology, University of Florida College of Medicine – Jacksonville, Jacksonville, USA

**Keywords:** surgery, treatment, management, brain metastases, leptomeningeal carcinomatosis, ovarian cancer

## Abstract

Leptomeningeal carcinomatosis (LMC) is an extremely rare site for metastasis from a primary ovarian cancer. LMC occurs when the thin layers of tissue that surround the brain and spinal cord are infiltrated by ovarian cancer metastasis. We present a case of a 63-year-old female with recurrent metastatic mucinous adenocarcinoma of the ovary who was diagnosed with LMC. While undergoing sixth-line chemotherapy, she presented with debilitating headaches and gait instability. Brain MRI revealed subarachnoid enhancement and other findings diagnostic of LMC. Given the rarity of this disease, treatment protocols have yet to be established. In patients with primary ovarian cancer that present with new onset neurological complaints, LMC should be suspected and appropriate imaging obtained.

## Introduction

Ovarian cancer is the leading cause of mortality among women with gynecologic cancer. The diagnosis of ovarian cancer is usually made during the later stages of the disease leading to the poor prognosis it carries [1]. Leptomeningeal carcinomatosis (LMC) is a rare site for metastasis from a primary ovarian cancer and accounts for less than 2% of all cases of brain metastases from ovarian cancer [2]. Treatment protocols for ovarian cancer have been widely studied and established [3], however, due to the lack of sufficient data, treatment protocols and subsequent outcomes for LMC have not been well established. Hence, there is heterogeneity in the approach to management with clinicians using their clinical judgment when managing patients with LMC [2]. Herein, we report a case of LMC from primary mucinous carcinoma of the ovary and briefly describe current literature, modalities of treatment and prognosis following the diagnosis.

## Case presentation

A 63-year-old female with a history of platinum-resistant recurrent metastatic mucinous adenocarcinoma of the ovary with peritoneal carcinomatosis, initially underwent standard surgical staging with total abdominal hysterectomy (TAH), bilateral salpingo-oophorectomy (BSO), pelvic lymphadenectomy (PLND), and omentectomy followed by six cycles of adjuvant platinum-based chemotherapy. A year later, her CA19-9 and CA-125 began to rise again, and CT imaging demonstrated metastatic disease along the liver margin and both subdiaphragmatic regions. She underwent a diagnostic laparoscopy, which demonstrated no definitive metastatic disease, however her liver was noted to be visually cirrhotic. Her disease progression was complicated by recurrent pleural effusion, requiring multiple thoracenteses and thoracotomy for removal of a loculated effusion. Her peritoneal carcinomatosis and omental implants continued to progress after initiation of sixth-line therapy with paclitaxel. While on her sixth line of therapy with single agent paclitaxel, she presented with unsteady gait, vomiting, and worsening daily debilitating headaches not relieved by medication for approximately one week. She was unable to further characterize her headache other than “debilitating”. She had experienced intermittent headaches throughout her clinical course, but she described her current headaches as different. Accompanying her headache was new onset weakness, dizziness, and nausea. Bloodwork was collected to rule out other common causes of her symptoms and the results were unremarkable. The only abnormality was a borderline low magnesium. Given the high index of suspicion for brain metastasis considering her relatively normal blood work in the setting of metastatic ovarian cancer MRI was scheduled for later the same day.

**Figure 1:**
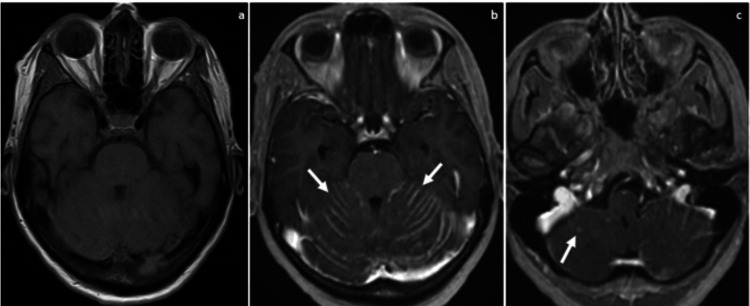
MRI of the brain. Pre-contrast T1-weighted sequence (a) demonstrating no hyperintense signal within the cerebellum. Post-contrast T1-weighted sequence (b) showing leptomeningeal enhancement within the cerebellar folia (white arrows). Post-contrast T1-weighted sequence (c) showing nodular small foci of enhancement (white arrow) concerning for solid intraparenchymal metastasis.

An MRI of her brain showed areas of enhancement in the subarachnoid spaces concerning for LMC, including diffusely along the cerebellar folia (with areas of adjacent edema in the cerebellum) (Figure 1). There were also areas of enhancement in the bilateral internal auditory canals and partially coating the surface of the brainstem and superior visualized cervical spinal cord. The patient began palliative brain radiation and received 3000cGy in 10 fractions. The patient subsequently died of her disease two weeks later.

## Discussion

After the confirmation of an LMC diagnosis, the median survival time is typically around 60 days. In this particular case, the patient survived for only 14 days following her diagnosis. Serous ovarian cancer makes up most cases leading to brain metastasis from a primary ovarian neoplasm, which accounts for the most common subtype of ovarian cancer leading to leptomeningeal disease [2]. To our knowledge, this is the first case being reported in the literature describing leptomeningeal disease resulting from ovarian cancer with mucinous histology. Mucinous histology, which accounts for around 3% of epithelial ovarian cancer [4], is thus a rare histological subtype in patients with LMC. The patient in this case presented with headaches, vomiting, and gait disturbances, which are the most common presenting symptoms of LMC. The new onset of her neurologic symptoms on the background of her mucinous ovarian carcinoma prompted further investigations. The diagnosis of LMC can be challenging due to the low sensitivity of various diagnostic modalities. The gold standard for diagnosis of leptomeningeal disease is documentation of malignant cells in CSF cytological examination [5]. However, after discussing the highly characteristic MRI findings and the likelihood that CSF cytology would not alter her management, the patient declined a lumbar puncture. In more recent times, MRI is now routinely used in the diagnosis of leptomeningeal carcinomatosis given improved high-quality MRI sequences and characteristic imaging findings [6]. The two most critical MRI sequences for leptomeningeal carcinomatosis are post-contrast T1 and fluid-attenuated inversion recovery (FLAIR) sequences [7]. Classically, leptomeningeal carcinomatosis presents as leptomeningeal enhancement on post-contrast T1 imaging and is best appreciated within the basal cisterns, folia of the cerebellum, gyral surfaces and around the cranial nerves [8]. FLAIR sequences will show serpiginous hyperintense signal within the sulci and subarachnoid spaces [9]. Additionally, enhancement around the cranial nerves is highly concerning for perineural spread, or metastasis, and may be the only imaging finding of leptomeningeal carcinomatosis in a subgroup of patients [8]. Other differentials for leptomeningeal enhancement and FLAIR hyperintense signal in the subarachnoid spaces include meningitis, subarachnoid hemorrhage, slow-flowing blood, high oxygen tension and propofol administration just prior to or during the MRI examination [10-12]. In patients with cancer and leptomeningeal enhancement on MRI, leptomeningeal carcinomatosis must be considered as the diagnosis until proven otherwise. Our case presents a classic example of MRI findings for leptomeningitis with subarachnoid and perineural enhancement (Figure 1).

Although a few cases of epithelial ovarian cancer leading to LMC have been reported (Table 1), the majority are small case studies; this limits the available data required to develop an optimal treatment regimen or approach.

**Table 1:** Review of literature regarding case reports of ovarian cancer leading to leptomeningeal metastasis. LMC: Leptomeningeal carcinomatosis; MTX: methotrexate; WBRT: whole-brain radiotherapy; (-): indicates data was not included.

Author	Age at Diagnosis	Presenting symptoms	Histology	Treatment after LMC diagnosis	Survival Time after LMC diagnosis (months)
Baek, Kubba [15]	66	cauda equina syndrome	papillary serous	intrathecal MTX, capecitabine & bevacizumab	8
Bangham et al. [16]	61	gait imbalance, trigeminal dysesthesia, perianal anesthesia, pedal anesthesia	BRCA-2 mutated, serous	oral chemotherapy	12
Bayas et al. [17]	56	intermittent diplopia, dysphagia. pedal paresthesia, generalized weakness	-	holistic therapy (patient request)	-
Bernstock et al. [18]	62	micturition difficulty, gait imbalance, headache, vision changes	malignant-mixed müllerian	intrathecal MTX	-
Cormio [19]	46	gait instability, dizziness, radicular pain	serous papillary	carboplatin, intrathecal MTX	15
Decelle et al. [20]	62	mood change, lethargy, nausea, headache, ataxia	-	oral steroids	1
Delord et al. [21]	57	paresthesia, deafness, vision changes, gait imbalance	-	intrathecal MTX	< 1
Erlap et al. [22]	36	headache, nausea, vomiting, vision changes, gait imbalance, altered consciousness	serous	topotecan, gemcitabine, docetaxel-carboplatin (in sequence)	24
Favier et al. [23]	54	headaches	-	WBRT and cisplatin	20
Gordon et al. [24]	49	headache, stiff neck, dizziness	serous	intrathecal MTX, oral chemotherapy, steroids, WBRT	6
Goto et al. [25]	60	gait instability, dizziness, nausea, temporal headaches	serous	intrathecal methotrexate, ommaya reservoir	18
Kahn et al. [26]	32	facial and upper extremity paresthesia	serous	carboplatin, paclitaxel, zoledronic acid	3
Kawagoe et al. [27]	55	dizziness, back pain, headache	serous	oral steroids	1.5
Khalil et al. [28]	54	radicular pain, headache, vertigo	papillary-serous	intrathecal MTX, WBRT	15
Koyuncuer et al. [5]	58	lower extremity weakness, speech disorders	clear cell	WBRT	-
Li et al. [29]	60	headache, vomiting	serous	WBRT	-
Melichar et al. [30]	-	seizure, headache, gait instability, cognitive impairment	-	intrathecal MTX, paclitaxel, oral steroids	1
Miller et al. [31]	49	diplopia, dysarthria, facial droop, nystagmus, lower extremity weakness, hand tremors	-	intrathecal topotecan, WBRT	4
Mukhopadhyay et al. [32]	58	headache, nausea, vomiting	papillary-serous	ommaya reservoir, MTX	1
Patel et al. [33]	56	facial numbness, mouth and eyelid drooping	serous	intrathecal MTX, WBRT	4
Sereno Moyano et al. [34]	-	memory issues, apraxia, headaches, nausea, vomiting		WBRT	1
Stein et al. [35]	55	seizures, headache, speech difficulties	serous papillary	oral steroids	<1
Stopa et al. {2}	67	headaches, dizziness, confusion	serous	surgical resection, cyberknife radiation	4
Tahir et al. [36]	58	headache, nausea, vomiting, vision changes, syncope, neck pain		intrathecal MTX	<1
Vitaliani et al. [37]	59	deafness, vertigo, imbalance	-	supportive	<1
Yamakawa et al. [38]	58	syncope, headache	serous	intrathecal MTX, oral steroids	7

Management of patients with LMC focuses on improving the neurologic symptoms, quality of life, and prolonging survival while at the same time minimizing toxicity of definitive treatment options. The use of systemic chemotherapy, whole-brain radiotherapy, intrathecal chemotherapy, and surgical resection alone or in combination has been the mainstay of treatment in the reported literature.

In 2020, a single-arm, open-label phase 2 trial of pembrolizumab in patients with solid tumor malignancies and LMC showed an improvement in three-month survival, however, the sample size was small [13]. Chen et al. examined 19 patients treated at a cancer center in the United States between 1985 and 2002 for ovarian cancer brain metastases. They found that patients who underwent gamma knife radiosurgery (GKRS) in addition to whole brain radiotherapy, resection, or chemotherapy had a median survival of 23 months, compared to four months for those who did not receive GKRS [14]. Due to the rarity of LMC and the resulting limitation in conducting robust treatment trials, a standardized management protocol is yet to be established. In the case of our patient, she began receiving palliative radiation to the brain shortly after the diagnosis of LMC. Unfortunately, she died of her disease just 14 days after the diagnosis. Leptomeningeal metastasis is known to indicate a poor prognosis, similar to our findings in this case. This case highlights the critical importance of early consideration and investigation of LMC in patients with ovarian cancer who exhibit new-onset neurologic symptoms or deficits. Such vigilance is crucial not only for early diagnosis but also for tailoring appropriate management strategies. Our patient's experience contributes to the growing body of evidence that underscores the need for prompt and targeted interventions in cases of suspected LMC, thereby potentially improving outcomes and informing future treatment protocols.

## Conclusions

In summary, we present a 63-year-old female with a history of platinum-resistant recurrent metastatic mucinous adenocarcinoma of the ovary with peritoneal carcinomatosis. While on sixth-line chemotherapy she presented to her pre-chemotherapy clinic visit with complaints of new-onset neurological symptoms. Promptly an MRI was performed and demonstrated classic findings associated with leptomeningeal disease and the patient began palliative brain radiation. The patient ultimately died two weeks after her initial diagnosis of leptomeningeal carcinomatosis.

Leptomeningeal carcinomatosis secondary to ovarian cancer, as illustrated by the case in this study, is linked to a poor prognosis. Despite utilizing multiple therapeutic approaches such as radiation, intravenous, and intrathecal chemotherapy, effective management remains a significant challenge. Emerging treatments like immunotherapy and GKRS offer potential, yet their impact on survival is still under investigation. This case report underscores the urgent need for more extensive research and larger-scale studies to enhance our understanding and development of optimal treatment strategies. Particularly, future research should focus on improving patient outcomes and quality of life. Our findings highlight the critical need for heightened clinical awareness in patients with primary ovarian cancer and prompt investigation in suspected cases to improve the prognosis and care for these patients.

## References

[1] Batool A, Kasi A (2023). Leptomeningeal carcinomatosis. StatPearls.

[2] Stopa BM, Cuoco JA, Adhikari S, Grider DJ, Rogers CM, Marvin EA (2022). Iatrogenic leptomeningeal carcinomatosis following craniotomy for resection of metastatic serous ovarian carcinoma: a systematic literature review and case report. Front Surg.

[3] Borella F, Bertero L, Morrone A (2020). Brain metastases from ovarian cancer: current evidence in diagnosis, treatment, and prognosis. Cancers (Basel).

[4] Rojas V, Hirshfield KM, Ganesan S, Rodriguez-Rodriguez L (2016). Molecular characterization of epithelial ovarian cancer: implications for diagnosis and treatment. Int J Mol Sci.

[5] Koyuncuer A, Varol E, Serarslan Yağcioğlu B, Bükte Y, Sakcı Z (2023). Cerebrospinal fluid-dissemination of a ovarian clear cell carcinoma: a leptomeningial carcinomatosis with diagnostic challenges. Diagn Cytopathol.

[6] Clarke JL, Perez HR, Jacks LM, Panageas KS, Deangelis LM (2010). Leptomeningeal metastases in the MRI era. Neurology.

[7] Singh SK, Leeds NE, Ginsberg LE (2002). MR imaging of leptomeningeal metastases: comparison of three sequences. AJNR Am J Neuroradiol.

[8] Singh SK, Agris JM, Leeds NE, Ginsberg LE (2000). Intracranial leptomeningeal metastases: comparison of depiction at FLAIR and contrast-enhanced MR imaging. Radiology.

[9] Tsuchiya K, Katase S, Yoshino A, Hachiya J (2001). FLAIR MR imaging for diagnosing intracranial meningeal carcinomatosis. AJR Am J Roentgenol.

[10] Nabavizadeh SA (2018). Application of 3D T1 black-blood imaging in the diagnosis of leptomeningeal carcinomatosis: potential pitfall of slow-flowing blood. AJNR Am J Neuroradiol.

[11] Anzai Y, Ishikawa M, Shaw DW, Artru A, Yarnykh V, Maravilla KR (2004). Paramagnetic effect of supplemental oxygen on CSF hyperintensity on fluid-attenuated inversion recovery MR images. AJNR Am J Neuroradiol.

[12] McKinney AM, Chacko Achanaril A, Knoll B, Nascene DR, Gawande RS (2018). Pseudo-leptomeningeal contrast enhancement at 3T in pediatric patients sedated by propofol. AJNR Am J Neuroradiol.

[13] Brastianos PK, Lee EQ, Cohen JV (2020). Single-arm, open-label phase 2 trial of pembrolizumab in patients with leptomeningeal carcinomatosis. Nat Med.

[14] Chen PG, Lee SY, Barnett GH, Vogelbaum MA, Saxton JP, Fleming PA, Suh JH (2005). Use of the Radiation Therapy Oncology Group recursive partitioning analysis classification system and predictors of survival in 19 women with brain metastases from ovarian carcinoma. Cancer.

[15] Baek WS, Kubba SV (2008). Cauda equina syndrome due to leptomeningeal carcinomatosis of the ovary. Gynecol Oncol.

[16] Bangham M, Goldstein R, Walton H, Ledermann JA (2016). Olaparib treatment for BRCA-mutant ovarian cancer with leptomeningeal disease. Gynecol Oncol Rep.

[17] Bayas A, Kondramashin A, Waheeds S, Swerdloff MA (2022). Ovarian adenocarcinoma with leptomeningeal metastases. Cureus.

[18] Bernstock JD, Ostby S, Fox B (2019). Cauda equina syndrome in an ovarian malignant-mixed müllerian tumor with leptomeningeal spread. Clin Case Rep.

[19] Cormio G, Loizzi V, Selvaggi L (2007). Leptomeningeal involvement after remission of brain metastases from ovarian cancer. Int J Gynaecol Obstet.

[20] Decelle L, D'Hondt L, Andre M (2007). Ovarian cancer associated with carcinomatous meningitis: a case report and review of the literature. Int J Gynecol Cancer.

[21] Delord JP, Fizazi K, el Hajj M, Pautier P, Lhommé C (1998). Isolated leptomeningeal metastasis from ovarian carcinoma: an unusual event. Eur J Cancer.

[22] Eralp Y, Saip P, Aydin Z, Berkman S, Topuz E (2008). Leptomeningeal dissemination of ovarian carcinoma through a ventriculoperitoneal shunt. Gynecol Oncol.

[23] Favier L, Truc G, Boidot R, Bengrine-Lefevre L (2020). Long-term response to Olaparib in carcinomatous meningitis of a BRCA2 mutated ovarian cancer: a case report. Mol Clin Oncol.

[24] Gordon AN, Kavanagh JJ, Wharton JT, Rutledge FN, Obbens EA, Bodey GP (1984). Successful treatment of leptomeningeal relapse of epithelial ovarian cancer. Gynecol Oncol.

[25] Goto Y, Katsumata N, Nakai S (2008). Leptomeningeal metastasis from ovarian carcinoma successfully treated by the intraventricular administration of methotrexate. Int J Clin Oncol.

[26] Kahn RM, Gandhi SK, Mvula MR, Li X, Frey MK (2020). Metastatic epithelial ovarian cancer to Meckel's cave with leptomeningeal spread at time of diagnosis. Gynecol Oncol Rep.

[27] Kawagoe Y, Nakayama T, Matuzawa S (2018). Epithelial ovarian carcinoma associated with metastases to central nervous system: two case reports. Case Rep Obstet Gynecol.

[28] Khalil AM, Yamout BI, Tabbal SD, Salem ZM, Mroueh AM (1994). Case report and review of literature: leptomeningeal relapse in epithelial ovarian cancer. Gynecol Oncol.

[29] Li HK, Harding V, Williamson R, Blagden S, Gabra H, Agarwal R (2012). Cerebral sinus thrombosis and leptomeningeal carcinomatosis in a patient with ovarian cancer. J Clin Oncol.

[30] Melichar B, Tomsová M, Rehák S, Malírová E, Simonová G (2008). Meningeal carcinomatosis as a late complication of brain metastases of epithelial ovarian carcinoma. Eur J Gynaecol Oncol.

[31] Miller E, Dy I, Herzog T (2012). Leptomeningeal carcinomatosis from ovarian cancer. Med Oncol.

[32] Mukhopadhyay S, Mukhopadhyay S, El-Zammar O, Khurana KK, Graziano SL (2006). CASE 2. Meningeal metastases from ovarian carcinoma. J Clin Oncol.

[33] Patel KB, Gaidis A, Stephens A, Thompson TZ, Williams H, Rungruang B (2018). A report of Bell's Palsy triggered by leptomeningeal metastases from recurrent high grade serous ovarian cancer. Gynecol Oncol Rep.

[34] Sereno Moyano M, Casado Saenz E, Belda Iniesta C, González Barón M (2005). Subacute dementia as presenting feature of carcinomatous leptomeningeal metastases. Clin Transl Oncol.

[35] Stein M, Steiner M, Ben-Schachar M (1987). Leptomeningeal involvement by epithelial ovarian carcinoma: a case report. Gynecol Oncol.

[36] Tahir N, Ram A, Jain N, Vemireddy LP, Zahra F (2021). Leptomeningeal carcinomatosis in epithelial ovarian cancer: a diagnostic challenge. Cureus.

[37] Vitaliani R, Spinazzi M, Del Mistro AR, Manara R, Tavolato B, Bonifati DM (2009). Subacute onset of deafness and vertigo in a patient with leptomeningeal metastasis from ovarian cancer. Neurol Sci.

[38] Yamakawa H, Ariga H, Enomoto A, Netsu S, Suzuki Y, Konno R (2009). Meningeal dissemination from an ovarian carcinoma with effective response to intrathecal chemotherapy. Int J Clin Oncol.

